# Primary cardiac sarcoma: reports of two cases and a review of current literature

**DOI:** 10.1186/1749-8090-2-34

**Published:** 2007-07-24

**Authors:** Mohan P Devbhandari, Shaista Meraj, Mark T Jones, Isaac Kadir, Ben Bridgewater

**Affiliations:** 1 Department of Cardiothoracic Surgery, Wythenshawe Hospital,Manchester,UK. M23 9LT

## Abstract

Primary cardiac sarcomas are rare tumors with an unfavourable prognosis. Complete surgical resection is currently the only mode of therapy proven to show any benefit. We report the cases of two patients presenting with features of obstruction and embolism and a presumed diagnosis of left atrial myxoma. At operation they were unexpectedly found to have large tumours raising strong suspicions of malignancy. Due to the extensive involvement of intracardiac structures with little possibility of reconstruction together with poor general condition of the patient, debulking was deemed to be the only viable option. Subsequent histology confirmed the diagnosis of sarcoma in both patients. Surgery produced immediate and effective symptom relief. The first patient died four months after the operation and second patient is still alive at 12 months after her operation. A brief review of literature on cardiac sarcoma is presented.

## Background

Primary cardiac tumours are rare [1] with an incidence of 0.0017 to 0.019%. Seventy-five percent of them are benign mostly myxomas and 25% are malignant predominantly consisting of sarcomas [2,3]. While tumours of vascular origin such as angiosarcoma are the commonest sarcoma of the heart, tumors of all cell lines including bony, neurogenic and soft tissue sarcomas have been reported to arise de novo from the heart. It is important to note that metastatic tumours in the heart are 20 to 30 times commoner than primary tumours [4]. Because of non-specificity of symptoms and rarity of these tumours they are often difficult to diagnose preoperatively and are missed occasionally. The advent of modern investigative tools including transesophageal echocardiogram, CT scan and cardiac MRI increases the likelihood of preoperative diagnosis. The majority are presumed to be benign myxoma and the suspicion of sarcomas is only made at the time of operation due to its invasive nature. Due to rarity of these tumours and lack of large representative case series, there is no uniform approach to treating these patients, and the benefits of adjuvant therapy are unclear [5]. We report on two cases of cardiac sarcoma treated in our hospital with a brief review of current literature.

## Case presentations

### Case 1

**Figure 1:**
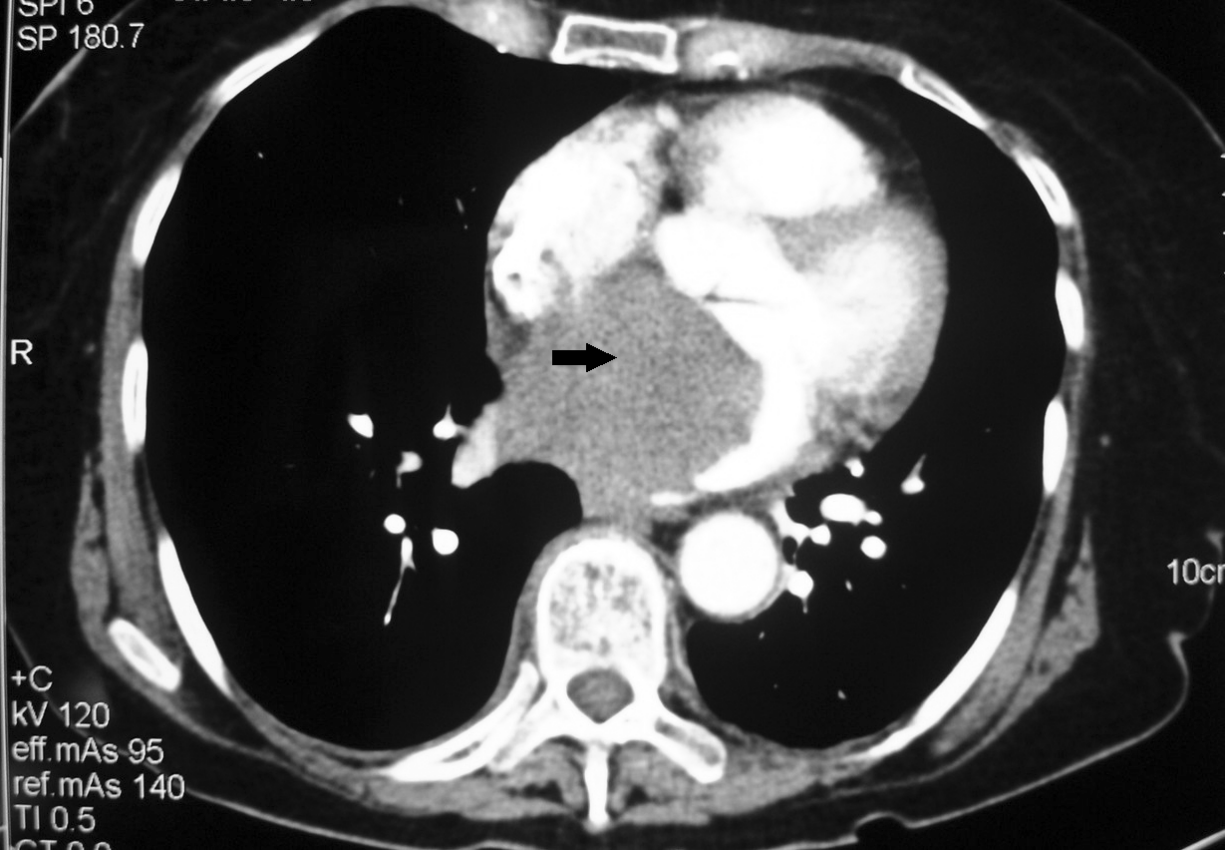
CT scan shows a large filling defect (black arrow) in the left atrium.

An 84-year-old lady presented with 6 weeks history of acute onset dyspnoea, lethargy and repeated haemoptysis. She had a previous history of chronic atrial fibrillation, ischemic heart disease, osteoarthritis and deep vein thrombosis. Clinical examination, blood tests and chest x ray were unremarkable. Her echocardiogram showed a large 4.2 × 5 cm echogenic mass in left atrium with moderate mitral regurgitation. Coronary angiography showed single vessel disease affecting the right coronary artery. A CT scan of the chest ruled out pulmonary embolism. A provisional diagnosis of left atrial myxoma or a thrombus was made and patient taken to theatre for an urgent surgery. The entire left atrial cavity was found to be filled with jelly like mass, which was diffusely infiltrating the left atrial wall. A curative resection was deemed impossible and debulking was performed. The patient made an uneventful recovery with significant improvement of symptoms. Histology confirmed the tumour to be undifferentiated pleomorphic sarcoma [figure 1]. Due to her advanced age and frailty she was not considered to be a suitable candidate for further treatment. Four months later she developed a metastatic pathological fracture of left femur requiring internal fixation. She developed severe bronchopneumonia postoperatively and succumbed to it. Her relatives declined post mortem examination.

### Case 2

A 37-year-old lady presented with 3 months history of right-sided pleuritic chest pain, high fever, night sweats and dry cough. She was extremely breathless with poor oxygenation and her blood tests showed presence of anaemia. Her chest x ray showed an ill-defined shadow on the right side of her chest. Despite treatment with broad-spectrum antibiotic for presumed chest infection her symptoms deteriorated. A contrast CT revealed cardiomegaly and a filling defect in the left atrium extending through the left ventricle. There was patchy non-specific shadowing in the right upper and middle lobes and a pleural effusion. A trans-thoracic echocardiogram confirmed a large 4.5 × 5 cm left atrial mass filling the entire left atrium and prolapsing into the left ventricle through the mitral valve.

**Figure 2:**
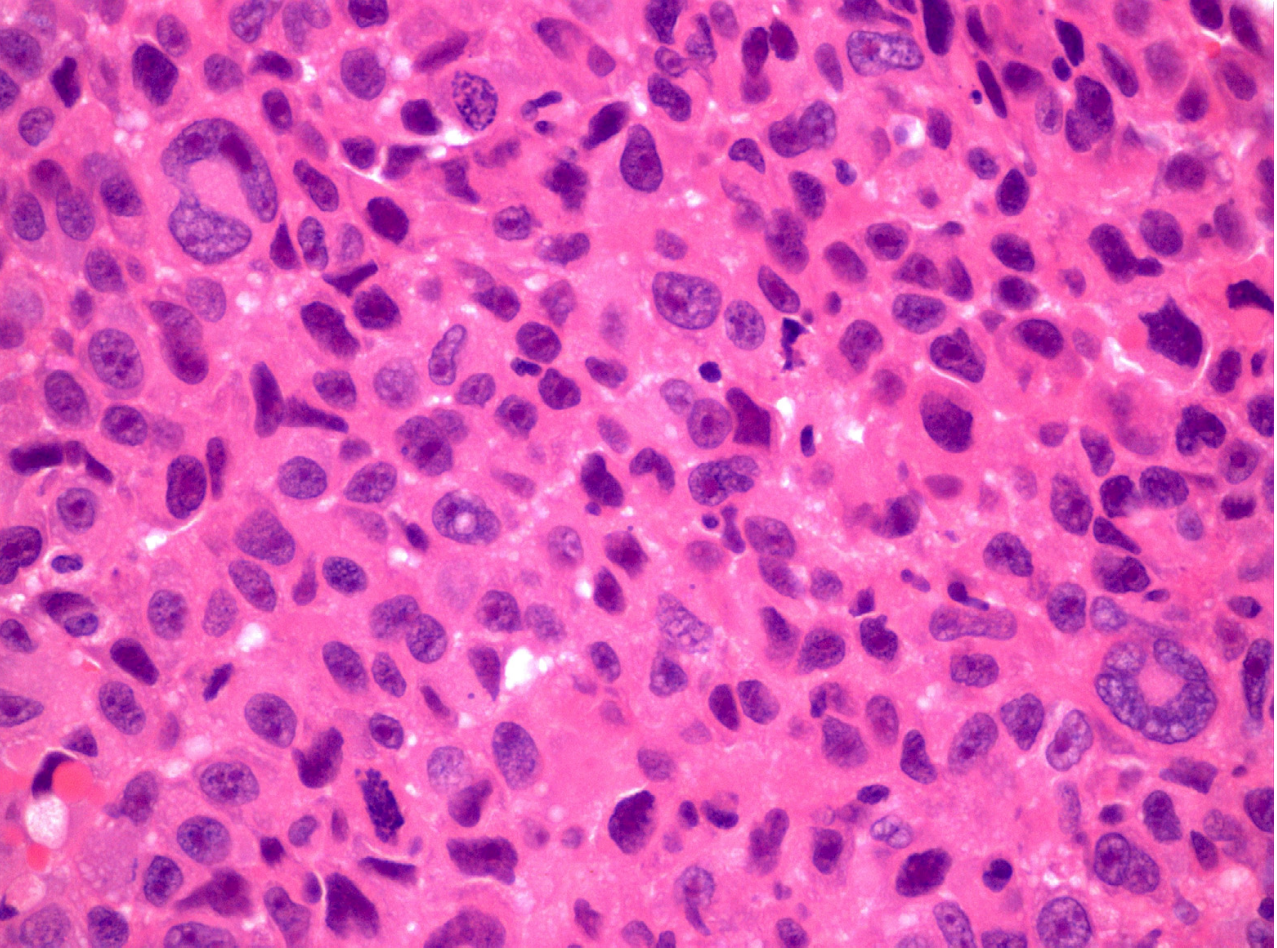
Histology showing appearance of sarcoma.

She continued to deteriorate and was extremely breathless at surgical presentation. She was taken to theatre with presumptive diagnosis of atrial myxoma. At surgery a large tumour was found arising from the left atrial side of the atrial septum at the confluence of the right superior pulmonary vein and superior vena cava. It was gelatinous in consistency and had a wide base. It was partly extending into the right superior pulmonary vein. The tumour was resected as completely as possible and the left atrium was reconstructed with a patch of pericardium. Postoperatively she made an uneventful recovery and was discharged home after a week. Definitive histology confirmed the tumour to be a myxoid sarcoma [Figure 2]. She underwent radiotherapy locally to chest but she declined chemotherapy. She is still alive twelve months after the operation. Her surveillance CT scan has not shown any evidence of local or distant recurrence.

## Discussion

Cardiac sarcomas are uncommon tumours. Approximately 25% of primary cardiac tumours are malignant and of these about 75% are sarcomas. In a large reported series angiosarcoma was the commonest (37%) followed by malignant fibrous histiocytoma (MFH) 24%, leiomyosarcoma 9%, rhabdomyosarcoma 7%, unclassified 7%, others 16% [5]. Angiosarcoma tends to be commoner in the right atrium and ventricle and MFH is commoner on the left side. These highly malignant tumours rapidly infiltrate all layers of the heart and metastasise widely. Hence up to 80% of patients have evidence of metastasis at the time of presentation [4].

The mean age of presentation is around 40 years [6] with no sex predilection. Rarely it can present in infancy and childhood [7]. Patients present after variable periods of symptoms which are often non-specific, ranging from few weeks to several months and almost all are symptomatic at presentation. The symptoms depend on the location and the extent of tumour. They manifest by one of the four mechanisms namely: obstruction to blood flow and valvular function, local invasion causing arrhythmia or pericardial effusion, embolism or systemic symptoms of dyspnoea, fever malaise and weight loss [8]. Dyspnea is the commonest symptom 60%, followed by chest pain 28%, congestive cardiac failure 28%, palpitations 24%, fever 14%, myalgia 10%, embolism 5% and constitutional symptoms of weakness, fever, anaemia [5].

Echocardiography by transthoracic or preferably transesophageal route is a convenient, rapid and inexpensive tool to identify intracardiac masses. A number of features have been reported that differentiate sarcomas from benign myxoma [9]. They are: 1) non-septal origin of mass, 2) extension into the pulmonary vein, 3) multiple masses, 4) a broad attachment on the left atrial wall, and 5) semisolid consistency. As illustrated by our cases and others [9] the picture is not easy to interpret and true diagnosis is only made as a unexpected finding at the operating table. Even if the diagnosis had been confirmed beforehand in our cases this still would not have changed our management, as surgery would still have been the best means of symptom relief.

The CT and MRI scans of chest and abdomen are complementary to echocardiography. They have the advantage of showing the extracardiac extent of tumour and presence of metastases in addition to the anatomical details of the lesion. CT scan shows myocardial infiltration, compression of cardiac chambers along with pericardial and great vessel involvement. MRI is useful to assess tumour volume, tumour burden, mediastinal invasion and response to therapy. Chest x ray may show cardiomegaly, infiltrates suggestive of congestive cardiac failure, pleural effusion, lung nodules, cardiac mass or left hemidiaphragm paralysis. Electrocardiogram is usually non-diagnostic being normal or may show non-specific changes such as conduction block right ventricular hypertrophy, atrial fibrillation, paroxysmal atrial tachycardia etc. A thorough search is mandatory for signs of metastasis, as up to 80% may have systemic metastasis at diagnosis, most commonly in the lung. Cytology of fine needle aspiration and pericardial biopsies for pericardial infiltration have not shown very accurate results. Transvenous endomyocardial biopsy is helpful to yield a histological confirmation before operation [10].

The role of chemotherapy or radiotherapy in the treatment of primary cardiac sarcoma has not proven to be beneficial and complete surgical excision is the only mode of therapy that has been shown to prolong survival [5]. Complete resection is possible in up to55% of cases where involvement is limited to atrial septum, small part of ventricle or valve. Additionally surgery also has a role in palliative resections for relief of symptoms, biopsy to confirm diagnosis or repeated resections. The operative mortality for surgical resection of cardiac sarcoma is high albeit acceptable at about 8.3% [5]. If malignancy is suspected or confirmed and the lesion appears anatomically resectable, with no evidence of spread, a complete resection should be considered [2]. Though the initial results of surgery are encouraging their long-term survival remains poor mostly due to local or systemic recurrence. Never the less surgery has been shown to prolong the survival and improve the quality of life.

The role of adjuvant chemotherapy remains undefined though there are some reports of adriamycin [5] and doxorubicin [7] showing some improvement in survival. Poor tolerance of radiation by the heart has limited the use of radiotherapy. Its role remains unproven though it has been used for treatment of positive margins after resections, palliation of aggressive localized disease and local or isolated distant recurrence. Gowdamarajan and Michler have reviewed reports of 21 patients who underwent transplantation for inoperable cardiac malignancy with overall mean survival or 12 months [11]. The concern about this mode of treatment is the possibility that immunosuppression may stimulate further tumour growth or recurrence and new neoplasia. In the current era of increasingly severe scarcity of donor organs this is unlikely to be a popular choice. This mode of treatment is advisable only for selected cases of widely resected non-angiosarcoma tumours with no obvious distant disease. Recently Reardon and colleagues have described successful tumour resection by explantation of the heart, complete tumour excision and then re-implantation in native position leading to long-term survival [12] in a small series of patients. This is difficult to reproduce in daily practice by non-transplant cardiac surgery centres. Where clinical suspicion is present preoperatively, these patients may be better served by referral to a designated national centre with expertise and interest in treatment of cardiac malignancies.

The completeness of surgical resection depends on the location of the tumour, extent of involvement of the myocardium and histology. In a reported series patients with complete resection were found to have median survival of 24 months compared to overall median survival of 10 months in all treated patients [5]. Overall actuarial survival was 14% at 24 months with median survival of 11 months. Prolonged survival of 10 years after a complete excision of left atrium rhabdomyosarcoma has been reported previously. Factors associated with increased survival after a resection of cardiac sarcoma are a left-sided location, mitotic rate less than 10 per high power field and absence of necrosis on histology [6]. Predictors of long-term mortality include NYHA class III and IV symptoms. Most of the patients ultimately die of metastasis or heart failure.

## Conclusion

High index of suspicion is required to achieve timely preoperative diagnosis of these patients. Wide surgical excision remains the only proven therapy which improves the symptoms and offers the potential for long term survival in selected patients. Role of adjuvant treatment still remains undefined.

## Competing interests

The author(s) declare that they have no competing interests.

## Authors' contributions

MD conceived of the paper and wrote the manuscript. SM followed up the patients and collected data. MJ reviewed the paper and contributed to designing of paper. IK and BB performed the operations and contributed to the content of the paper. All authors read and approved the final manuscript.
